# *N*′-(4-Hy­droxy­benzyl­idene)-2-methyl­benzohydrazide

**DOI:** 10.1107/S1600536810035063

**Published:** 2010-09-04

**Authors:** Chun-Bao Tang

**Affiliations:** a Department of Chemistry, Jiaying University, Meizhou 514015, People’s Republic of China

## Abstract

The title hydrazone compound, C_15_H_14_N_2_O_2_, was prepared by the condensation of 4-hy­droxy­benzaldehyde with 2-methyl­benzohydrazide in methanol. The dihedral angle between the two benzene rings is 42.3 (2)°. In the crystal structure, mol­ecules are linked by inter­molecular O—H⋯O, O—H⋯N and N—H⋯O hydrogen bonds, forming a three-dimensional framework.

## Related literature

For general background to hydrazones, see: Rasras *et al.* (2010 ▶); Pyta *et al.* (2010 ▶); Angelusiu *et al.* (2010 ▶); Fun *et al.* (2008 ▶); Singh & Singh (2010 ▶); Ahmad *et al.* (2010 ▶). For bond-length data, see: Allen *et al.* (1987 ▶).
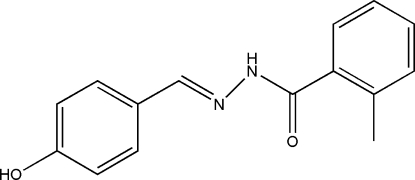


## Experimental

### 

#### Crystal data

C_15_H_14_N_2_O_2_
*M* *_r_* = 254.28
Orthorhombic, 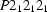

*a* = 7.6900 (15) Å
*b* = 11.701 (2) Å
*c* = 14.471 (3) Å
*V* = 1302.1 (4) Å^3^
*Z* = 4
Mo *K*α radiation
μ = 0.09 mm^−1^
*T* = 298 K
0.20 × 0.20 × 0.18 mm

#### Data collection

Bruker SMART CCD area-detector diffractometer
Absorption correction: multi-scan (*SADABS*; Sheldrick, 1996 ▶) *T*_min_ = 0.983, *T*_max_ = 0.984
10755 measured reflections
1634 independent reflections
1502 reflections with *I* > 2σ(*I*)
*R*_int_ = 0.024

#### Refinement

*R*[*F* ^2^ > 2σ(*F* ^2^)] = 0.036
*wR*(*F* ^2^) = 0.101
*S* = 1.12
1634 reflections
177 parameters
1 restraint
H atoms treated by a mixture of independent and constrained refinement
Δρ_max_ = 0.15 e Å^−3^
Δρ_min_ = −0.23 e Å^−3^

### 

Data collection: *SMART* (Bruker, 2002 ▶); cell refinement: *SAINT* (Bruker, 2002 ▶); data reduction: *SAINT*; program(s) used to solve structure: *SHELXS97* (Sheldrick, 2008 ▶); program(s) used to refine structure: *SHELXL97* (Sheldrick, 2008 ▶); molecular graphics: *SHELXTL* (Sheldrick, 2008 ▶); software used to prepare material for publication: *SHELXTL*.

## Supplementary Material

Crystal structure: contains datablocks global, I. DOI: 10.1107/S1600536810035063/ci5176sup1.cif

Structure factors: contains datablocks I. DOI: 10.1107/S1600536810035063/ci5176Isup2.hkl

Additional supplementary materials:  crystallographic information; 3D view; checkCIF report

## Figures and Tables

**Table 1:** Hydrogen-bond geometry (Å, °)

*D*—H⋯*A*	*D*—H	H⋯*A*	*D*⋯*A*	*D*—H⋯*A*
O1—H1⋯O2^i^	0.82	1.96	2.7657 (18)	166
O1—H1⋯N1^i^	0.82	2.52	2.995 (2)	118
N2—H2⋯O1^ii^	0.91 (1)	2.14 (1)	2.995 (2)	158 (2)

Symmetry codes: (i) 
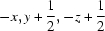
; (ii) 
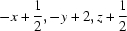
.

**Fig. 1.**
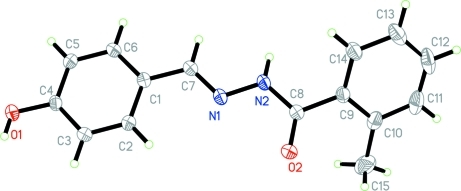
The molecular structure of the compound, showing the atom-numbering scheme. Displacement ellipsoids are drawn at the 30% probability level.

**Fig. 2.**
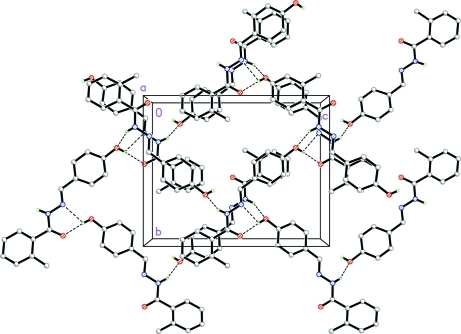
Molecular packing of the title compound, with hydrogen bonds shown as dashed lines.

## supplementary crystallographic information

### Comment

Hydrazone compounds have been received much attention in biological chemistry and structural chemistry in the last few years (Rasras *et al.*, 2010; Pyta *et al.*, 2010; Angelusiu *et al.*, 2010; Fun *et al.*, 2008; Singh & Singh, 2010; Ahmad *et al.*, 2010). In the present paper, the author reports the crystal structure of the title new hydrazone compound (Fig. 1).

In the title molecule, the dihedral angle between the two benzene rings is 42.3 (2)°. The torsion angles C1—C7—N1—N2, C7—N1—N2—C8 and N1—N2—C8—C9 are 2.9 (2), 0.9 (2), and 0.2 (2)°, respectively. All the bond lengths are within normal values (Allen *et al.*, 1987).

In the crystal structure of the compound, molecules are linked through O–H···O, O–H···N, and N–H···O intermolecular hydrogen bonds (Table 1), forming a three-dimensional network (Fig. 2).

### Experimental

4-Hydroxybenzaldehyde (0.1 mmol, 12.2 mg) and 3-methylbenzohydrazide (0.1 mmol, 15.0 mg) were dissolved in methanol (20 ml). The mixture was stirred at reflux for 10 min to give a clear colourless solution. Colourless block-shaped crystals of the compound were formed by slow evaporation of the solvent over several days.

### Refinement

Atom H2 was located in a difference Fourier map and refined isotropically, with the N–H distance restrained to 0.90 (1) Å [*U*_iso_(H) = 0.08 Å^2^]. Other H atoms were constrained to ideal geometries, with C–H = 0.93–0.96 Å, O–H = 0.82 Å, and with *U*_iso_(H) = 1.2*U*_eq_(C) and 1.5*U*_eq_(C15 and O1). In the absence of significant anomalous dispersion effects, Friedel pairs were averaged.

### Crystal data

**Table:**

C_15_H_14_N_2_O_2_	*F*(000) = 536
*M**_r_* = 254.28	*D*_x_ = 1.297 Mg m^−^^3^
Orthorhombic, *P*2_1_2_1_2_1_	Mo *K*α radiation, λ = 0.71073 Å
Hall symbol: P 2ac 2ab	Cell parameters from 4917 reflections
*a* = 7.6900 (15) Å	θ = 2.2–27.1°
*b* = 11.701 (2) Å	µ = 0.09 mm^−^^1^
*c* = 14.471 (3) Å	*T* = 298 K
*V* = 1302.1 (4) Å^3^	Block, colourless
*Z* = 4	0.20 × 0.20 × 0.18 mm

### Data collection

**Table:**

Bruker SMART CCD area-detector diffractometer	1634 independent reflections
Radiation source: fine-focus sealed tube	1502 reflections with *I* > 2σ(*I*)
graphite	*R*_int_ = 0.024
ω scans	θ_max_ = 27.0°, θ_min_ = 2.2°
Absorption correction: multi-scan (*SADABS*; Sheldrick, 1996)	*h* = −9→9
*T*_min_ = 0.983, *T*_max_ = 0.984	*k* = −14→14
10755 measured reflections	*l* = −18→18

### Refinement

**Table:**

Refinement on *F*^2^	Primary atom site location: structure-invariant direct methods
Least-squares matrix: full	Secondary atom site location: difference Fourier map
*R*[*F*^2^ > 2σ(*F*^2^)] = 0.036	Hydrogen site location: inferred from neighbouring sites
*wR*(*F*^2^) = 0.101	H atoms treated by a mixture of independent and constrained refinement
*S* = 1.12	*w* = 1/[σ^2^(*F*_o_^2^) + (0.0604*P*)^2^ + 0.1042*P*] where *P* = (*F*_o_^2^ + 2*F*_c_^2^)/3
1634 reflections	(Δ/σ)_max_ = 0.001
177 parameters	Δρ_max_ = 0.15 e Å^−^^3^
1 restraint	Δρ_min_ = −0.23 e Å^−^^3^

### Special details

**Table:**

Geometry. All esds (except the esd in the dihedral angle between two l.s. planes) are estimated using the full covariance matrix. The cell esds are taken into account individually in the estimation of esds in distances, angles and torsion angles; correlations between esds in cell parameters are only used when they are defined by crystal symmetry. An approximate (isotropic) treatment of cell esds is used for estimating esds involving l.s. planes.
Refinement. Refinement of F^2^ against ALL reflections. The weighted R-factor wR and goodness of fit S are based on F^2^, conventional R-factors R are based on F, with F set to zero for negative F^2^. The threshold expression of F^2^ > 2sigma(F^2^) is used only for calculating R-factors(gt) etc. and is not relevant to the choice of reflections for refinement. R-factors based on F^2^ are statistically about twice as large as those based on F, and R- factors based on ALL data will be even larger.

### Fractional atomic coordinates and isotropic or equivalent isotropic displacement parameters (Å^2^)

**Table:**

	*x*	*y*	*z*	*U*_iso_*/*U*_eq_	
N1	0.1045 (2)	0.76789 (13)	0.47260 (10)	0.0442 (4)	
N2	0.1260 (2)	0.70443 (13)	0.55210 (10)	0.0433 (4)	
O1	0.1276 (2)	1.15917 (11)	0.16838 (8)	0.0439 (3)	
H1	0.0660	1.1323	0.1275	0.066*	
O2	0.0277 (2)	0.54745 (11)	0.47842 (8)	0.0480 (4)	
C1	0.1310 (2)	0.94736 (15)	0.39752 (11)	0.0369 (4)	
C2	0.0694 (2)	0.90743 (16)	0.31255 (12)	0.0397 (4)	
H2A	0.0288	0.8328	0.3080	0.048*	
C3	0.0675 (2)	0.97611 (15)	0.23590 (12)	0.0391 (4)	
H3	0.0264	0.9482	0.1799	0.047*	
C4	0.1276 (2)	1.08777 (15)	0.24249 (11)	0.0345 (4)	
C5	0.1884 (3)	1.12934 (16)	0.32573 (13)	0.0416 (4)	
H5	0.2283	1.2042	0.3300	0.050*	
C6	0.1899 (3)	1.05962 (16)	0.40250 (12)	0.0427 (4)	
H6	0.2309	1.0880	0.4584	0.051*	
C7	0.1410 (3)	0.87360 (15)	0.47796 (12)	0.0402 (4)	
H7	0.1747	0.9043	0.5345	0.048*	
C8	0.0859 (2)	0.59215 (15)	0.54852 (11)	0.0375 (4)	
C9	0.1113 (2)	0.52835 (15)	0.63685 (12)	0.0389 (4)	
C10	0.1715 (3)	0.41533 (17)	0.63644 (15)	0.0483 (5)	
C11	0.1871 (3)	0.3609 (2)	0.7213 (2)	0.0631 (7)	
H11	0.2286	0.2863	0.7229	0.076*	
C12	0.1439 (3)	0.4126 (3)	0.80218 (18)	0.0703 (8)	
H12	0.1559	0.3732	0.8576	0.084*	
C13	0.0828 (3)	0.5227 (2)	0.80235 (14)	0.0649 (7)	
H13	0.0519	0.5579	0.8576	0.078*	
C14	0.0678 (3)	0.5808 (2)	0.71959 (13)	0.0498 (5)	
H14	0.0280	0.6559	0.7194	0.060*	
C15	0.2224 (4)	0.3530 (2)	0.5498 (2)	0.0763 (8)	
H15A	0.1196	0.3306	0.5167	0.114*	
H15B	0.2886	0.2863	0.5656	0.114*	
H15C	0.2915	0.4024	0.5116	0.114*	
H2	0.190 (3)	0.733 (2)	0.5994 (13)	0.080*	

### Atomic displacement parameters (Å^2^)

**Table:**

	*U*^11^	*U*^22^	*U*^33^	*U*^12^	*U*^13^	*U*^23^
N1	0.0618 (10)	0.0404 (8)	0.0303 (7)	−0.0046 (8)	−0.0064 (7)	0.0072 (6)
N2	0.0622 (10)	0.0384 (8)	0.0294 (7)	−0.0076 (8)	−0.0103 (7)	0.0068 (6)
O1	0.0610 (8)	0.0385 (6)	0.0322 (6)	−0.0012 (6)	−0.0033 (6)	0.0082 (5)
O2	0.0697 (9)	0.0413 (7)	0.0329 (6)	−0.0064 (7)	−0.0078 (6)	−0.0010 (6)
C1	0.0426 (9)	0.0353 (8)	0.0327 (8)	0.0014 (8)	−0.0021 (7)	0.0036 (7)
C2	0.0499 (10)	0.0322 (8)	0.0371 (9)	−0.0036 (8)	−0.0030 (8)	0.0023 (7)
C3	0.0485 (10)	0.0373 (9)	0.0316 (8)	0.0001 (8)	−0.0038 (7)	−0.0014 (7)
C4	0.0380 (8)	0.0335 (8)	0.0319 (8)	0.0054 (7)	0.0010 (7)	0.0054 (7)
C5	0.0537 (11)	0.0304 (8)	0.0407 (9)	−0.0019 (8)	−0.0045 (8)	0.0012 (7)
C6	0.0566 (11)	0.0393 (9)	0.0322 (8)	−0.0018 (9)	−0.0088 (8)	−0.0008 (8)
C7	0.0504 (10)	0.0396 (9)	0.0305 (8)	−0.0009 (8)	−0.0039 (8)	0.0016 (8)
C8	0.0437 (9)	0.0370 (8)	0.0317 (8)	−0.0007 (8)	−0.0010 (7)	0.0020 (7)
C9	0.0414 (9)	0.0390 (9)	0.0361 (8)	−0.0069 (8)	−0.0055 (8)	0.0068 (7)
C10	0.0468 (11)	0.0388 (9)	0.0592 (12)	−0.0054 (8)	−0.0076 (9)	0.0087 (9)
C11	0.0553 (13)	0.0510 (12)	0.0831 (17)	−0.0073 (10)	−0.0172 (13)	0.0303 (13)
C12	0.0643 (14)	0.0870 (18)	0.0594 (14)	−0.0195 (15)	−0.0179 (11)	0.0431 (14)
C13	0.0711 (15)	0.0877 (19)	0.0361 (10)	−0.0117 (14)	−0.0034 (10)	0.0141 (11)
C14	0.0590 (12)	0.0541 (11)	0.0364 (9)	−0.0040 (10)	−0.0023 (9)	0.0065 (9)
C15	0.092 (2)	0.0484 (12)	0.0880 (18)	0.0146 (14)	−0.0047 (17)	−0.0092 (14)

### Geometric parameters (Å, °)

**Table:**

N1—C7	1.271 (2)	C6—H6	0.93
N1—N2	1.379 (2)	C7—H7	0.93
N2—C8	1.350 (2)	C8—C9	1.493 (2)
N2—H2	0.907 (10)	C9—C14	1.387 (3)
O1—C4	1.3594 (19)	C9—C10	1.401 (3)
O1—H1	0.82	C10—C11	1.389 (3)
O2—C8	1.226 (2)	C10—C15	1.503 (3)
C1—C6	1.391 (3)	C11—C12	1.358 (4)
C1—C2	1.398 (2)	C11—H11	0.93
C1—C7	1.451 (2)	C12—C13	1.371 (4)
C2—C3	1.370 (2)	C12—H12	0.93
C2—H2A	0.93	C13—C14	1.382 (3)
C3—C4	1.389 (3)	C13—H13	0.93
C3—H3	0.93	C14—H14	0.93
C4—C5	1.381 (2)	C15—H15A	0.96
C5—C6	1.378 (2)	C15—H15B	0.96
C5—H5	0.93	C15—H15C	0.96
			
C7—N1—N2	116.54 (15)	O2—C8—C9	122.87 (16)
C8—N2—N1	117.68 (14)	N2—C8—C9	115.07 (15)
C8—N2—H2	120.6 (17)	C14—C9—C10	120.08 (17)
N1—N2—H2	119.9 (17)	C14—C9—C8	119.10 (17)
C4—O1—H1	109.5	C10—C9—C8	120.77 (17)
C6—C1—C2	118.12 (15)	C11—C10—C9	117.2 (2)
C6—C1—C7	120.18 (16)	C11—C10—C15	119.6 (2)
C2—C1—C7	121.65 (16)	C9—C10—C15	123.19 (19)
C3—C2—C1	121.31 (16)	C12—C11—C10	122.5 (2)
C3—C2—H2A	119.3	C12—C11—H11	118.8
C1—C2—H2A	119.3	C10—C11—H11	118.8
C2—C3—C4	119.52 (16)	C11—C12—C13	120.2 (2)
C2—C3—H3	120.2	C11—C12—H12	119.9
C4—C3—H3	120.2	C13—C12—H12	119.9
O1—C4—C5	118.15 (16)	C12—C13—C14	119.3 (2)
O1—C4—C3	121.59 (15)	C12—C13—H13	120.3
C5—C4—C3	120.26 (15)	C14—C13—H13	120.3
C6—C5—C4	119.83 (16)	C13—C14—C9	120.7 (2)
C6—C5—H5	120.1	C13—C14—H14	119.7
C4—C5—H5	120.1	C9—C14—H14	119.7
C5—C6—C1	120.96 (16)	C10—C15—H15A	109.5
C5—C6—H6	119.5	C10—C15—H15B	109.5
C1—C6—H6	119.5	H15A—C15—H15B	109.5
N1—C7—C1	121.21 (16)	C10—C15—H15C	109.5
N1—C7—H7	119.4	H15A—C15—H15C	109.5
C1—C7—H7	119.4	H15B—C15—H15C	109.5
O2—C8—N2	122.01 (15)		

### Hydrogen-bond geometry (Å, °)

**Table:**

*D*—H···*A*	*D*—H	H···*A*	*D*···*A*	*D*—H···*A*
O1—H1···O2^i^	0.82	1.96	2.7657 (18)	166
O1—H1···N1^i^	0.82	2.52	2.995 (2)	118
N2—H2···O1^ii^	0.91 (1)	2.14 (1)	2.995 (2)	158 (2)

Symmetry codes: (i) −*x*, *y*+1/2, −*z*+1/2; (ii) −*x*+1/2, −*y*+2, *z*+1/2.
